# The Use of an FR1 Schedule Operant Approach-Avoidance Paradigm to Measure the Aversiveness of Neuropathic and Inflammatory Pain

**DOI:** 10.3389/fpain.2021.793958

**Published:** 2022-01-20

**Authors:** Celina A. Salcido, Cassie M. Argenbright, Tiffany Aguirre, Alex D. Trujillo, Perry N. Fuchs

**Affiliations:** ^1^University of the Incarnate Word School of Osteopathic Medicine, San Antonio, TX, United States; ^2^Department of Psychology, University of Texas at Arlington, Arlington, TX, United States

**Keywords:** approach, avoidance, inflammation, neuropathy, pain, operant

## Abstract

Pain is a subjective, private, yet universal phenomenon that depends on a unique combination of sensory, affective, and evaluative characteristics. Although preclinical models have been used to understand much of pain physiology, the inability to communicate with animals limits affective and evaluative feedback and has constrained traditional behavioral methods to adequately represent and study the multidimensional pain experience. Therefore, this study sought to characterize the affective component of pain within a novel operant approach-avoidance paradigm (AAP) to determine which type of pain (inflammatory and neuropathic) may be more aversive. To reveal the possible differences in pain aversiveness within the AAP paradigm, animals received bilateral inflammatory and neuropathic pain conditions and were given the choice to a) forgo appetitive reward by not receiving noxious stimulus of either inflammatory or neuropathic conditions or b) receive noxious stimulus in exchange for an appetitive reward. Although all pain conditions produced significant hypersensitivity, the AAP results revealed there was no preference in the stimulation of a specific paw in the bilateral pain conditions. The finding suggests that despite unique clinical pain characteristics for inflammatory and neuropathic conditions, the lack of observable differences in the pain conditions may not necessarily equate to the overall similarity in aversiveness, but rather that the fixed ratio (FR1) paradigm presentation allowed appetitive reward to be more salient, highlighting the complexities of competing motivational drives of pain and hunger when satiating hunger is always guaranteed. Thus, future studies should seek to further tease apart this relationship with a different schedule and food-controlled methodologies. The development of such preclinical approaches can thoroughly investigate the intricacy of competing drives and likely reveal important information regarding the complexity of pain, enhancing our understanding of pain perception in individuals suffering from comorbid pain states.

## Introduction

The challenge of pain stems directly from its subjectivity and has proven difficult in not only treatment, but even in producing an accurate definition of what it is (1–3). Because pain is perceived only by the sufferer, the specification of pain features *via* verbal report has been used in clinical realms to convey the experience of pain (4–6). Although seemingly trivial and common in practice, identifying semantically descriptive properties has led to a promising characterization of pain since the specific language used is goal-directed and is used to convince others that pain is indeed perceived and needs to be resolved (7–11). One of the most widely accepted and validated tests for measuring clinical pain is the McGill Pain Questionnaire (7, 12, 13). This measurement is effective in such a way that it provides information from all three dimensions of pain such that differences in each modality can be assessed using the language provided by the sufferer. In fact, Melzack (14) assessed phantom limb (neuropathic pain syndrome) and arthritis (chronic inflammatory disease) according to words chosen and reported that although relatively similar, there were differences in the Pain Rating Index (PRI) such that the sensory and affective components of pain were relatively lower for arthritic individuals than patients with a phantom limb (14). Beyond this, there has been a very little direct comparison of types of pain. Instead, the focus has been primarily on looking at the similarities and differences of physiological mechanisms.

Despite challenges in communicating pain in preclinical realms (15), the shared properties of nociception of humans and non-humans including anatomy, physiology, and behavior along with the historical understanding of pain as a purely sensory modality have allowed sensory reflexive information to dominate pain research (1, 2, 16, 17). Although reflexive behaviors such as innate withdrawal, licking, elevating. have provided critical information regarding causal neural processing of painful stimuli, they are predominately concentrated on the elucidation of peripheral and spinal mechanisms associated with pain processing. Thus, the description of sensory physiology does not provide much information beyond stimulus parameters, failing to accurately portray the entire pain experience.

To further characterize the relevant assessments of pain processing similar to clinical realms, preclinical pain evaluations began to shift toward a more affective approach [(18–21)]. Since pain affect is comprised of the emotional understanding of pain's unpleasant and aversive qualities and the motivation to relieve pain, emotion, and motivation is tightly intertwined, where emotion, an interoceptive component, produces a behavioral motivation (21–23). To quantify this dimension, the use of non-reflexive measurements like an escape and avoidant behaviors have been interpreted as indicators of the unpleasantness of pain (3, 24). The justification arises from the assumption that since pain is aversive, an animal would be motivated to terminate pain. As a result, the pain would also provide a learning experience such that the animal would learn to avoid the painful environment (25). Various existing models for evaluating this component include conditioned place preference (26, 27), place escape/avoidance paradigm (19), two-temperature choice (28), and conditioned place aversion (29), which do so by allowing an animal to choose between environments associated with either a noxious or non-noxious stimulus. In particular, McNabb et al. (18) utilized a modified Place/Escape Avoidance Paradigm (mPEAP) box to compare affective processing in L5 spinal nerve ligation (SNL) and carrageenan conditions. Instead of the prototypical light/dark box implemented in the PEAP, the mPEAP designed a box with alternating white and black stripes that were horizontal on one side and vertical on the other to produce an unbiased approach by eliminating a preference such that neither side of the chamber is preferred. This allowed for a comparison of a simultaneous bilateral pain condition where an SNL was introduced to the left paw and a carrageenan injection was administered to the right paw. The results of the study revealed no preference in time spent on one side of the box over the other for bilateral conditions, whereas unilateral conditions preferred stimulation of the non-noxious paw (18). This finding suggested that pain affect may not differ across pain types in this assessment. Although this paradigm revealed important insight when comparing affective mechanisms of neuropathic and inflammatory pain, the model only allowed for escape and/or avoidance responses, which may not be always possible especially for homeostatic maintenance.

Although the research into the affective dimension of pain has been a critical enhancement, very little research has been directed toward understanding the cognitive dimension. Concurrent disruption of homeostasis from different stressors can promote competition between the drives [LaGraize et al., 2004; (30, 31)]. Approach-avoidance conflicts utilize this phenomenon to pose a unique situation associated with both reward and punishment such that an animal must choose which to approach and which to avoid. By assessing the value of costs and benefits to the available options, an animal will choose the most optimal outcome. Preclinical approach-avoidance assessments have previously utilized basic needs such as hunger and pain (32). In such cases, these studies have revealed that pain typically demands salience over hunger when animals are facing large appetitive rewards in exchange for pain. Thus, approach-avoidance studies such as this can provide evidence of hierarchical prioritization among conflicting drives. Although these studies have predominantly evaluated the relationship between hunger and pain, it may be possible to assess other conflicting drives among the simultaneous experience of two different subtypes of pain, such as inflammatory and neuropathic pain conditions (33).

Taken altogether, the research regarding the perceptual experience of different levels of pain affect seems to be ambiguous. Although some of the previous studies point to no apparent differences in the affective component of different pain conditions (i.e., neuropathic vs. inflammatory), this is counterintuitive to evidence from clinical reports suggesting differences in the physical, emotional, and cognitive qualities of unique pain conditions (7, 12, 14, 34). It is reasonable to predict that, based on the multidimensionality of pain and that certain pain states involve unique perceptual profiles, the sensory and affective modalities of the pain experience are also likely to differ across pain types. Therefore, the purpose of this study was to assess the aversive nature of the two most commonly used pain models utilizing a simplified version of one of our previous operant approaches (33). The present paradigm utilized a protocol such that animals were given the choice to (a) press the presented lever to receive an appetitive reward and simultaneous tactile stimulation in the paw associated with the unilateral intraplantar (Ipl) carrageenan condition, SNL, or their respective controls or (b) forgo appetitive reward by not receiving a noxious stimulus of either the inflammatory or neuropathic condition (i.e., Ipl carrageenan or SNL). We hypothesized that the aversive stimulus would be sufficient to punish operant responding as an indication of the affective/motivational dimension of pain. To investigate the suggestive differences in quantitative or qualitative aspects of these conditions, the fourth group of animals treated with both Ipl carrageenan and SNL on opposite hindlimbs was included. These results were explored by comparing paw withdrawal thresholds to the effects of tactile stimulation in the approach/avoidance paradigm (AAP) paradigm.

## Materials and Methods

### Animals and Procedures

Twenty-four adult male Sprague Dawley rats initially weighing between 176 and 200 g were purchased from Charles River, placed in a single housing in a separate colony room on a 12:12 dark/light cycle, and allowed to habituate for 3 days. Afterward, the animals were placed on a food-controlled diet with a variable time feeding schedule until 85% of the original weight is achieved. Water was provided *ad libitum*.

Once at 85% of the original weight, the animals were trained to lever-press for appetitive reward. The animals varied on the number of training days to ensure they reached the criteria for test day (80% response rate) but had a minimum of 7 training days. Training occurred once a day in standard operant chambers (Med Associates, Inc.) in which the animals were shaped to press a lever for appetitive reward (45 mg grain-based pellet) at variable times of the day to ensure expectation of appetitive reward could impact lever-pressing behavior. On baseline day and test day (3 days later), the animals were subjected to lever-press for appetitive reward in a modified operant box described further as the AAP. For all operant procedures, the animals were subjected to a fixed ratio (FR1) schedule within which appetitive reinforcement was provided after each behavioral response.

### Training Phases

**Figure 1:**
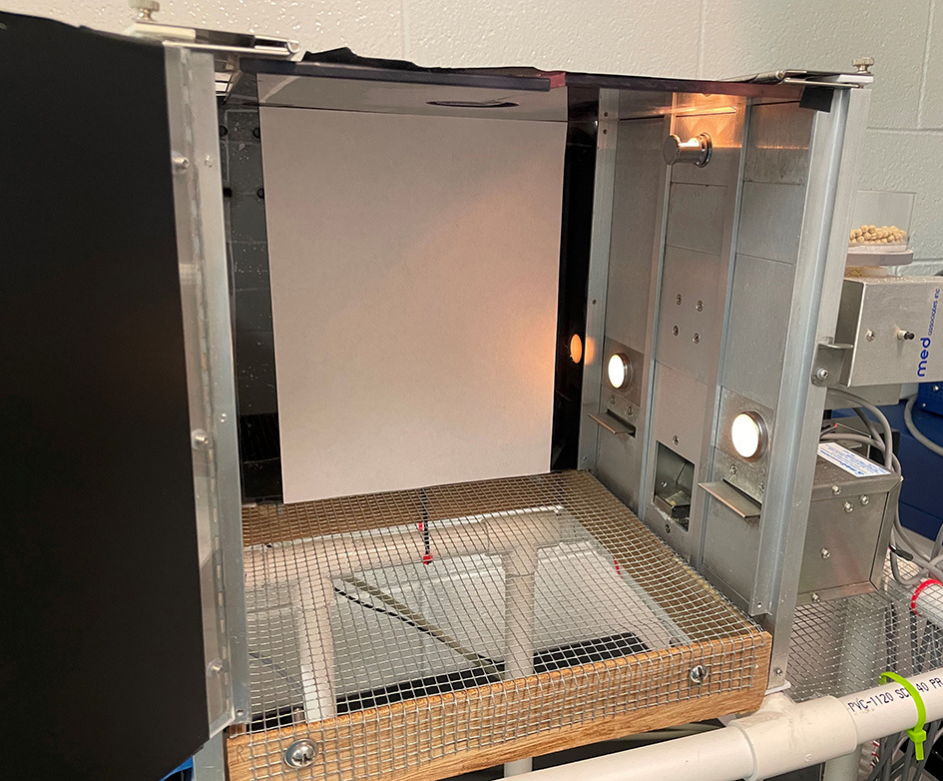
The Med Associates Inc. operant chamber used for the approach-avoidance paradigm (AAP), designed with a wire mesh floor and levers located on the left and right sides of the food dispenser. Above each lever is a single stimulus light that serves as a cue to signal the active lever.

All operant training occurred in a standard Med Associates Inc. operant chamber containing a wire mesh floor and levers located on the left and right sides of a food hopper, which dispenses appetitive rewards following lever-pressing or retraction of the lever. Above each of the levers is a single stimulus light that serves as a cue to signal the active lever and eventual stimulation associated with lever-presses during baseline and testing AAP (Figure 1).

Day one of training consisted of a manual training phase, where the animals were exposed to the paradigm. To begin, the stimulus light above the active lever was presented for 5 s, after which the lever below the light was presented and then retracted. At the initiation of the retraction of the lever, one pellet was dispensed. This sequence occurred every 30 s until 60 trials had been completed. Inclusion criteria to meet the next training phase occurred when the animals have successfully associated the lever retraction with appetitive reward, which was signified by the consumption of all 60 pellets from the training. This training occurred for both left and right levers where the animals starting with left training on day 1 would have to pass to begin day 2 with the right training and vice versa. Once both lever sides are associated with the consumption of 60 pellets, the animals could move to the second phase of training on day 3. If the animals failed to consume all 60 pellets, they would repeat the manual training phase 1 associated with the failed side the following day. The animals have 5 tries total to meet the criteria for the next round until they are excluded from the study.

On the second phase of training, the animals were subjected to additional manual training where the stimulus light indicating the active lever remained on for 5 s before the lever was presented. Afterward, the lever remained out for the animal to lever-press an unlimited number of times for a total of 30 min. If the animals were able to lever-press during this time, the lever retracted one pellet was dispensed, and the lever would immediately be presented again. The animals that had successfully lever-pressed for 40 times or more met the criteria and then moved onto the next phase of training. This training occurred on both left and right levers where on day 3 would be presented with left lever and if passed, moved on to day 4 training on the right lever. Once both lever sides met the criteria, the animals continued to automatic training on day 5. If the animals failed to lever-press 40 times, they would repeat the manual phase training 2 associated with the failed lever side the following day. The animals have 5 tries to meet criteria before exclusion.

In the third phase of training, the animals were subjected to automatic phase training. This was characterized by the presentation of the stimulus light above the lever that would be presented for 5 s. Afterward, the lever was presented for 10 s before the lever retracted back in. If the animal lever-pressed within the 10 s, a pellet was dispensed, the lever retracted back in, the lever light was turned off, and a timeout period of 25 s before the next trial was initiated. This occurred every 35 s, where the light was presented, the lever was presented, remained out until pressed or the time allotted 10 s ran out, and a timeout of 25 s for a total of 60 trials. The animals that had successfully lever-pressed for at least 80% of trials, i.e., lever-pressed for at least 48 out of the 60 trials or omitted no more than 12 trials, moved onto the next level of training. This training had to occur for both left and right levers such that left automatic training occurred on day 5 and if met the criteria, moved to right automatic training on day 6. If both days met the criteria, the animals moved on to dual training on day 7. If the animals did not meet the criteria, they would repeat the automatic training associated with the failed lever on the following day. The animals had 5 tries to meet the criteria before exclusion.

In the fourth training phase, the animals were subjected to dual training. This task modeled the automatic training, but both levers and associated stimulus lever lights were presented at the same time, providing a choice to the animal on which lever to press. This training continued until there was an unbiased lever preference as indicated by no more than 65% of lever-presses occurring at one single bar. The animals had to meet these criteria in addition to at least an 80% bar pressing success rate to move on to baseline AAP testing. If the animals did not meet the criteria, they would repeat the dual training on the following day. The animals had 5 tries to meet the criteria before exclusion.

On baseline day, the animals were placed into the AAP used on the testing day; however, no pain manipulations were present. To produce the AAP box, a standard operant chamber (Med Associates) was removed from the sound attenuating box hub and the steel rod floor was removed. The chamber was fixed atop a mesh floor PVC platform so that a mechanical stimulus could be applied to the plantar surface of the hind paws. The transparent outside walls of the chamber were covered using black contact paper to minimize the potential that movement outside of the chamber would impact the animal's behavior. Essentially, modifying an operant chamber allowed for mechanical stimulation of the plantar surface of the hind paws, despite the location of the animal in the apparatus. These modifications produced unrestricted access to the animal's paws, and thus a reduction in error related to delayed or omitted stimulation. In this phase, the lever was presented every 30 s and remained out for 10 s. Five seconds before the lever was presented, the light cue above the lever remained on and was used to determine which paw to stimulate in response to a lever-press. If the animal's lever was pressed, stimulation was initiated by the experimenter with a suprathreshold Von Frey filament (476 mN of force) to the associated paw (left lever, left paw or right lever, right paw) while the food pellet was dispensed. Thus, the animals could consume appetitive rewards right after lever-press and stimulation. Stimulation was applied with enough force to bend the filament once applied to the hind paw, and was withdrawn immediately once the force applied was enough to bend the filament. Assessing the behavior prior to testing conditions provided for a baseline assessment of behavior in the paradigm. Once the animals had successfully lever-pressed at least for 80% of trials, i.e., lever-pressed for at least 48 out of the 60 trials or omitted no more than 12 trials, they moved onto surgery and then test day.

The criteria for the animals to advance through the experimental protocol are described in Table 1.

**Table 1:** Criteria and procedure for approach-avoidance paradigm (AAP).

**Daily**	**Procedure**	**Inclusion criteria**
Weigh/feed	Food-controlled diet	Training begins at 85% of free access weight
Weigh/feed	Manual training phase 1 (MT1) (counterbalanced)	Must associate lever-presses with food reward in hopper
Weigh/feed	Manual training phase 2 (MT2) (counterbalanced)	Must achieve over 40 lever-presses
Weigh/feed	Automatic training (counterbalanced)	Must achieve 80% of presses
Weigh/feed	Dual train	Must achieve 80% of presses; 65% unbiased pressing
Weigh/feed	Baseline MPWT	No sensitivity in hind paws
Weigh/feed	Baseline AAP test	Set baseline for animals under normal conditions
Weigh/feed	L5 ligation or sham surgery	3 days recovery
Weigh/feed	Test day	
	Saline or carrageenan injection	
	Priming	Must lever-press for 10 trials
	AAP Test	
	mPEAP Test	
	Post MPWT (three hours after initial injection)	Ensure effectiveness of carrageenan and ineffectiveness of saline to sensitivity

### Mechanical Paw Withdrawal Testing

Once the criteria were reached and prior to pain manipulation, the animals were subjected to a mechanical paw withdrawal threshold testing (MPWT) prior to AAP testing to ensure no hypersensitivity of the hind paws. For this, the animals were placed into Plexiglas chambers, placed atop a mesh to access the hind paws for tactile stimulation, and left to habituate for 10 min. Tactile sensitivity was measured using the up/down method by stimulating the plantar portion of the hind paws using a set of Von Frey monofilaments (3.85, 5.68, 9.74, 18.39, 39.42, 77.3, 135.3, and 251.34 mN). Each trial of testing began with the 9.74 mN Von Frey filament delivered to the left hind paw for ~1 s, then to the right paw, or vice versa depending on the orientation of the animal. If no withdrawal response was observed (i.e., paw withdrawal or licking), the next highest force was used, whereas the next lowest force was delivered if a response was observed. This procedure was repeated until no response was made at the highest force (251.34 mN) or until five stimuli were administered in total. The 50% paw withdrawal threshold for each trial was calculated using the following formula: [Xth]log = [vFr]log + ky, where [vFr] was the force of the last Von Frey used, k = 0.2593 is the average interval (in log units) between the Von Frey monofilaments, and y was a value that depends upon the pattern of withdrawal responses. If an animal did not respond to the highest Von Frey monofilament (251.34 mN), then y = 1.00, and the 50% mechanical paw withdrawal response for that paw is calculated to be 456.63 mN (35). This test was conducted three times and the scores from each trial were averaged to determine the mean threshold to tactile stimulation for the right and left paws for each animal at both baselines prior to AAP testing and on test day after AAP testing.

### L5 Spinal Nerve Ligation Surgery

Once the animals advanced through operant training, they were subjected to either SNL (*n* = 12) or sham surgery (*n* = 12). To do so, the animals were put under isoflurane anesthesia (3% initiation and 2 % maintenance). For SNLs, ligation procedures followed the methods previously described by Kim and Chung (36). After shaving and skin preparation with the aseptic betadine, a 1–1.5-inch incision was made on the slightly left of the spinal cord. A portion of the transverse process was then removed to help to expose the L5 spinal nerve, which was dissected from the L6 spinal nerve and then tightly ligated using a 6–0 silk suture. After suturing of the muscle layer, the wound was closed using surgical staples. Sham surgeries were conducted in a similar fashion, apart from the removal of the transverse process and ligation of the nerve. The respiratory rate during the surgical procedure was closely monitored and recorded every 15 min on an operative sheet. Three full days of recovery were allotted before testing occurred, during which time postoperative health was closely monitored (eating, drinking, activity, respiratory function, chromodachyhorrea, wound status, rough hair coat).

### Test Day

On the test day, the animals were randomly assigned to receive a subcutaneous injection into the plantar surface of the right hind paw with either 5 ml of 1% carrageenan lambda (Sigma) to induce an acute inflammatory pain condition or normal saline (*n* = 12). The animals were then allowed to habituate for 3 h. An MPWT test was then performed 30 min later to ensure the effectiveness of carrageenan and SNL to induce hypersensitivity.

Immediately after MPWT, the animals were placed in the operant AAP to quantify the animal's approach/avoidance behavior associated with the presentation or lack thereof of training to the modified operantnoxious stimulation.

Before testing, the animals were subjected to a priming session, where they were presented both levers and had to successfully lever-press for 10 consecutive times without tactile stimulation of the Von Frey filament. This was done to ensure that the animals were able to transfer the performance of the task learned in the standard operant chamber used during lever-press training to the modified operant chamber and to also ensure that any non-evoked pain that the animals might have been experiencing did not impact simple lever-pressing performance. During the test session, a light cue above the lever was presented for 5 s and then a lever was presented for 10 s at 30-s intervals for a total of 60 trials. The animals were able to lever-press once during this 10 s interval, at which the lever retracted back in, one pellet was dispensed, and the 25-s timeout began. Thus, the single pressing of the lever signified the end of the trial. If the animal did not lever-press within the 10-s interval, the trial would end, it was considered an omission, and a new trial would commence in another 25 s. This allowed for a minimum of 0 pellets and a maximum of 60 pellets within the paradigm, in which the number of lever-presses correlated with the number of pellets received. Lever-presses for appetitive reward were immediately followed by mechanical stimulation of the paw using a suprathreshold (476 mN). Von Frey monofilament, while no lever responses resulted in no stimulation. The light cue was randomly presented 5 s before the lever was dispensed to indicate to the animal which foot was going to be stimulated, allowing for 30 sessions for each hind paw/lever combination. Therefore, this paradigm presents the animals with an approach-avoidance conflict such that they were presented with two behavioral choices: (1) press the lever and receive noxious stimulation to the associated paw or (2) not press the lever, avoid stimulation to the associated paw, and forego appetitive reward. Suppression of reward seeking was viewed as an indication of the unpleasantness of the noxious stimulation. The number of trials yielding a response as well as latencies to lever-press was recorded *via* MED-PC operant coding by Med Associates for each trial.

Therefore, the conditions were as followed: Sham/saline (*n* = 6); Sham/carrageenan (*n* = 6); SNL/saline (*n* = 6); SNL/carrageenan (n = 6).

### Statistical Analyses

Data analyses were performed using Statistical Package for the Social Sciences (SPSS). To analyze MPWT for the hind paws, a between-subjects ANOVA was used to assess the mean mechanical thresholds of pain (carrageenan and SNL). To analyze the percentage of trials that the animals pressed the lever for appetitive reward, an ANOVA was performed to evaluate group differences for left and right levers. Analysis of latency to press the lever for appetitive reward utilized a mixed repeated measures ANOVA with pain condition (carrageenan, SNL, saline, sham) as the between-subjects variable and time (baseline and test) as the within-subjects variable. All *post-hoc* analyses were assessed using Least Significant Difference (LSD).

## Results

### Mechanical Paw Withdrawal Threshold (MPWT) by Paw and Condition

#### Left Paw (SNL or Sham Condition)

**Figure 2:**
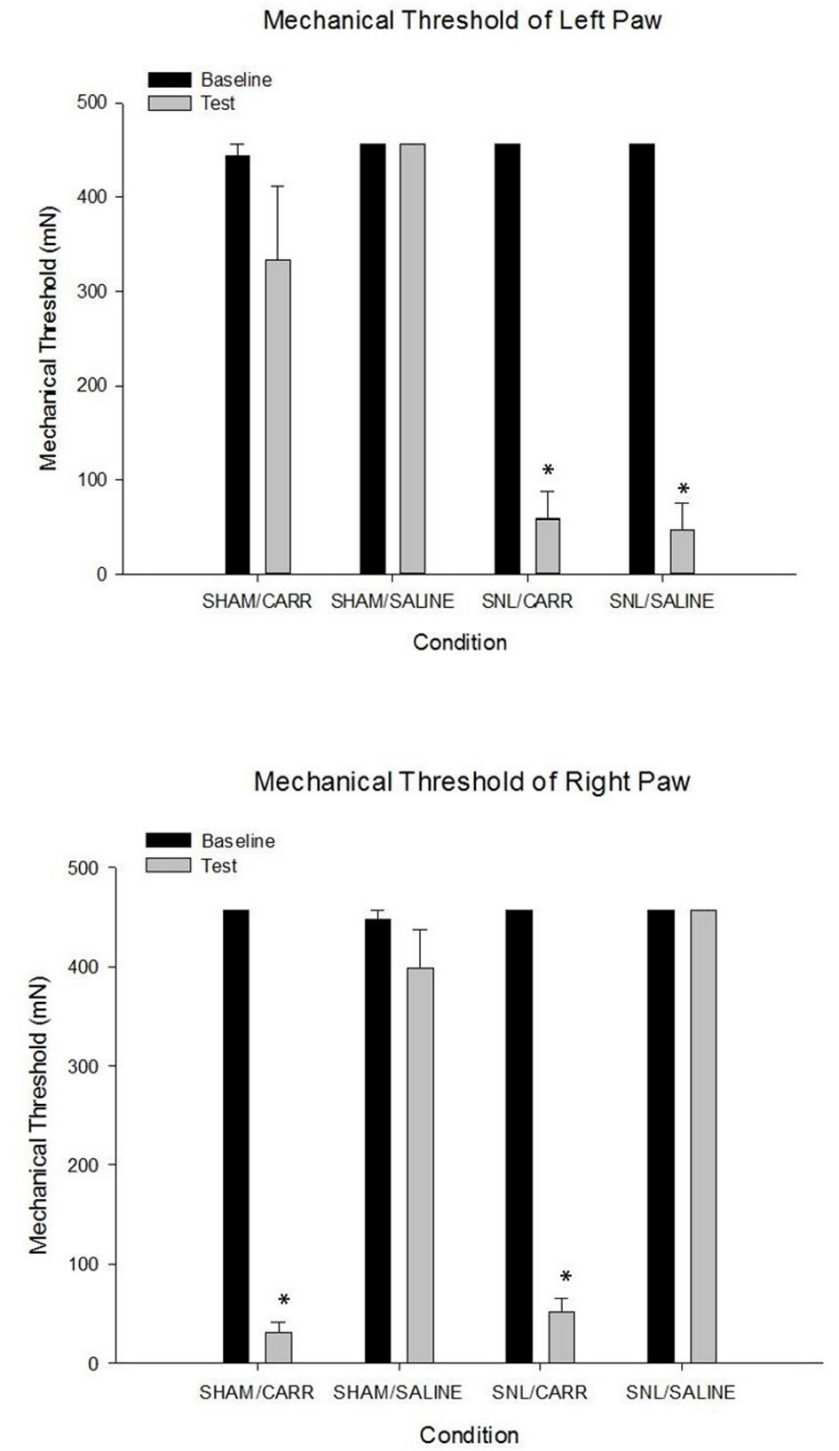
Mechanical paw withdrawal threshold (MPWT) by pain conditions for both left and right paws for baseline and test days using mean (±SEM) MWPT score for each condition. The left paw was associated with L5 spinal nerve ligation (SNL) or sham condition. SNL conditions were associated with a significant decrease in withdrawal threshold for test days compared to sham animals. The right paw was associated with carrageenan or saline condition. Carrageenan-treated conditions were associated with a significant decrease in the withdrawal threshold for test days compared to saline animals. *p < 0.001.

A one-way ANOVA was used to examine condition group differences in the left paw for baseline and test days to assess the efficacy of the SNL to induce neuropathic pain hypersensitivity. As expected, there was no significant main effect of pain condition at baseline, *F*_(3,20)_ = 1.00, *p* = 0.413. However, there was a significant main effect of the pain condition at testing, *F*_(3,20)_ = 21.294, *p* < 0.001, $\eta_{p}^{2}$ = 0.762. Additional analysis revealed that the animals in the sham/carrageenan group (*M* = 332.953, *SE* = 44.193) had a significantly higher MPWT than the animals in both the SNL/carrageenan condition (*M* = 58.60, *SE* = 44.193) and the SNL/saline condition (*M* = 46.752, *SE* = 44.193) on test day. The animals in the sham/saline condition (*M* = 456.630, *SE* = 44.193) also had a significantly higher MPWT on test day, than the animals in the SNL/carrageenan condition and the SNL/saline condition (Figure 2).

### Right Paw (Carrageenan or Saline Condition)

A one-way ANOVA was run to examine the condition group difference in the right paw across baseline and testing days to assess the efficacy of carrageenan to induce inflammatory pain hypersensitivity. As expected, there was no significant main effect of condition group at baseline, *F*_(3,20)_ = 1.00, *p* = 0.413. There was, however, a significant main effect of the condition group at testing, *F*_(3,20)_ = 111.521, *p* < 0.001, $\eta_{p}^{2}$ = 0.944. Specifically, the animals in the sham/carrageenan group (*M* = 31.597, *SE* = 21.217) had a significantly lower MPWT than the sham/saline group (*M* = 398.317, *SE* = 21.217). The animals in the sham/carrageenan also had a significantly lower MPWT than the animals in the SNL/saline group (*M* = 456.630, *SE* = 21.217). The animals in the sham/saline had a significantly higher MPWT than the animals in SNL/carrageenan (*M* = 52.138, *SE* = 21.217). The animals in the SNL/carrageenan group had a significantly lower MPWT than the animals in the SNL/saline group (Figure 2).

### AAP Lever-Presses by Condition

**Figure 3:**
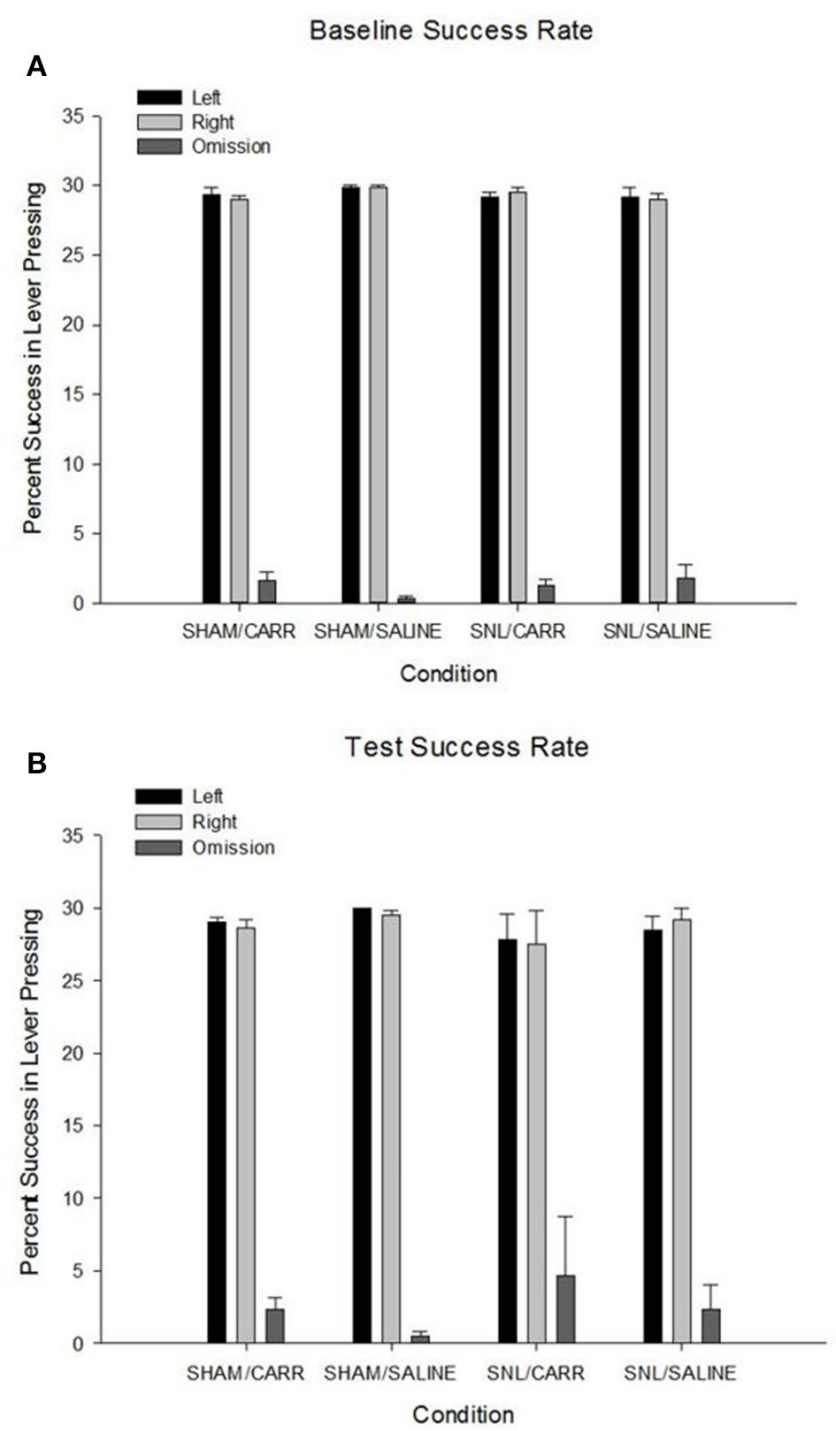
Percent success (±SEM) in lever-pressing for left and right levers and pooled left and right omissions across pain conditions for baseline **(A)** and test days **(B)**. Success rates were quantified by using an individual number of lever-presses for the paradigm on test day and dividing by the total number of trials for each side (30). There were no differences in success rates for lever-pressing or omissions to the AAP.

A one-way ANOVA was used to analyze differences in trial percentages of left and right lever-presses for an appetitive reward. There was no significant main effect of pain condition on left lever-presses at baseline, *F*_(3,20)_ = 0.501, *p* = 0.686, or on test day, *F*_(3,20)_ = 0.779, *p* = 0.519. The same pattern was observed for right lever-presses, with no significant main effect of condition at baseline, *F*_(3,20)_ = 1.622, *p* = 0.216., or on test day, *F*_(3,20)_ = 0.481, *p* = 0.699. Analysis of lever-press omissions revealed there was no significant main effect of pain condition found on number of omissions at baseline, *F*_(3,20)_ = 1.327, *p* = 0.294, or on test day, *F*_(3,20)_ = 0.578, *p* = 0.636 (Figure 3).

### Latency to Lever-Press by Condition

**Figure 4:**
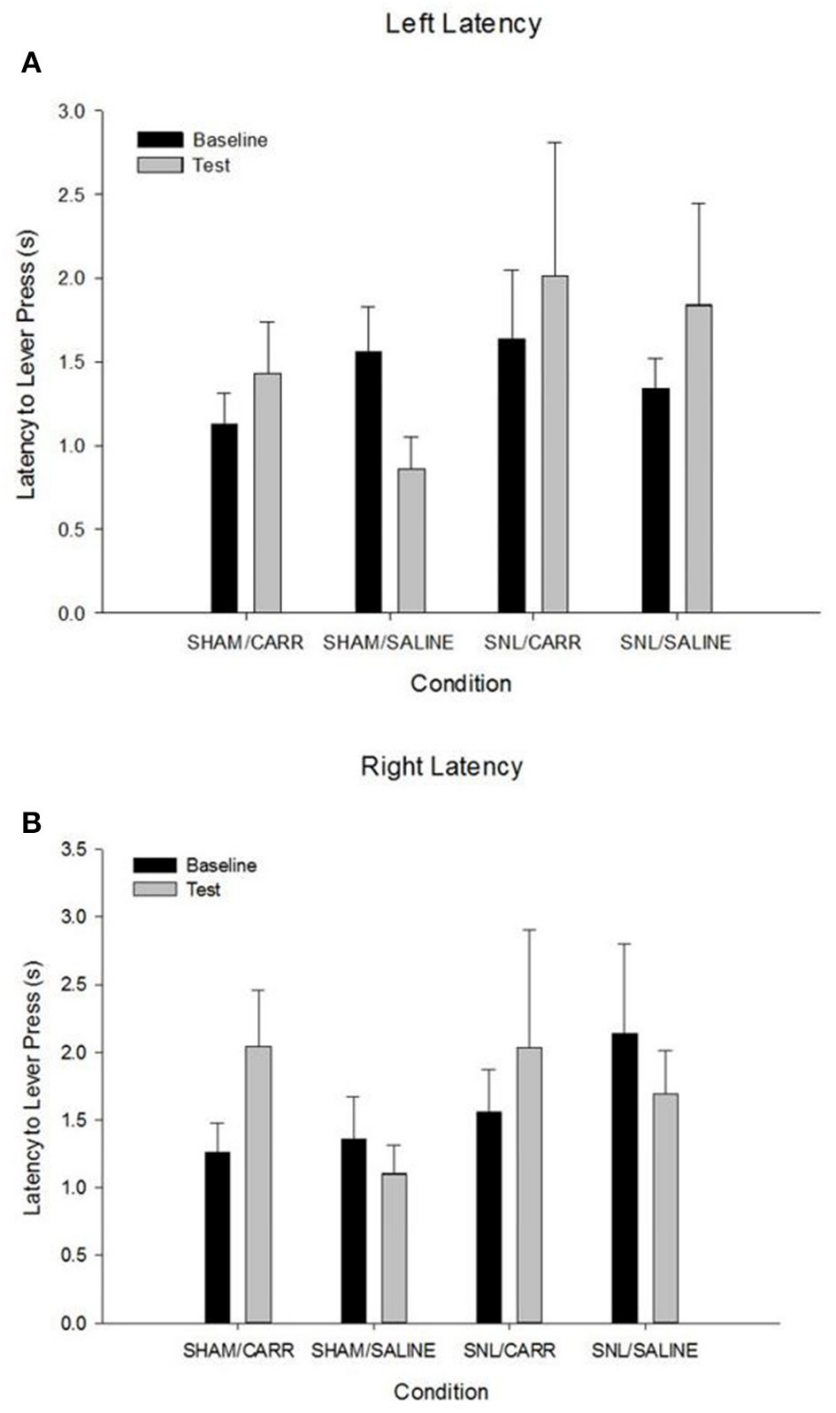
Average latency in seconds (±SEM) to respond to the lever by pressing for the appetitive reward for left and right levers across baseline **(A)** and test days **(B)**. There were no differences in latency to lever-press across conditions in the AAP.

To analyze the latency of lever-presses for an appetitive reward, a mixed model repeated measures ANOVA was used with pain condition as the between-subjects variable and time as the within-subjects variable. There was found to be no significant main effects of pain condition, *F*_(3,17)_ = 0.341, *p* = 0.796, or time, *F*_(1,17)_ = 0.068, *p* = 0.798. The analysis further revealed that there was no significant interaction effect between time and pain condition, *F*_(3,17)_ = 1.622, *p* = 0.222, on latency to lever-press (Figure 4).

## Discussions

Humans have the overwhelming privilege to be able to communicate their pain. In fact, the descriptors of pain can be effectively categorized into sensory/discriminative, affective/motivational, and cognitive/evaluative dimensions of pain (14, 17). Such descriptors can be used to help distinguish differences of subtypes of pain. Obviously, animals cannot convey pain with language, so researchers must develop an advanced behavioral methodology that is designed to evaluate the multidimensionality of various preclinical pain models. Operant conditioning seeks to shape behavior by its consequences, within the theory of a “three-term contingency,” such that a discriminative stimulus (SD) is presented to elicit a behavioral response (R), with the consequent stimulus (SC) being applied of the intention to alter the learning association between the two: SD → R → SC (37). The novel AAP presented animals with the extended lever, coupled with the visual light component and the auditory component of the lever extension (SD), to elicit the response of lever-pressing in exchange for an appetitive reward (R). The noxious tactile stimulation applied to the hind paw (SC) served to investigate any changes in prioritization of competing for motivational and cognitive drives as a result of the potential uniqueness in aversive qualities associated with carrageenan-induced inflammation or SNL-induced neuropathy. More simply, the paradigm allowed animals to “choose” a preference, thereby, allowing for the ability to quantify pain affect or aversiveness between the pain states. A preference for stimulation of one paw over the other could indicate a “perceptual difference” between pain conditions. Thus, the ultimate goal of the experimental protocol was to examine the relative magnitude of pain affect of inflammatory and neuropathic pain.

It was hypothesized that the pain conditions would be associated with a decrease in success rate and/or an increase in latency to lever-press due to consistent stimulus evoked nociception associated with every lever-press. The data from the AAP revealed no significant difference in *success rate* or *latency to lever-press* across the conditions. This outcome was not anticipated since pain processing increases decision latency in both humans (38) as well as animals [(30, 39, 40)]. It should be noted that there are several methodological differences among previous studies [(30, 41–47)] that could account for the data. For instance, differences in the magnitude and type of nociceptive stimulus (thermal, mechanical, chemical) would be expected to have an impact on behavioral outcomes, given that a more salient nociceptive stimulus could develop a stronger associative relationship with the reinforcer. The timing and duration of the response/reinforcer contingency as well as various schedules of reinforcement can also impact behavioral outcomes (37, 48–51). Due to the complexity of this specific paradigm, it is possible that the FR1 schedule did not allow us to tease apart the distinctive motivational and cognitive processes that might have been exhibited through a disruption in the drive to satiate hunger. It would be of interest to determine if distinguishing between schedules of reinforcement may impact success and latency to lever-press, thus contributing to teasing apart the intricate cognitive and motivational relationship associated with these types of nociceptive conditions (37). In addition, manipulating the timing of the evoked nociceptive stimulus relative to the food reinforcer should also be examined. The current protocol allowed animals to press the lever for food prior to an evoked nociceptive stimulus. Concurrent timing of the presentation of the food reinforcer and stimulus-evoked nociception parameters could result in a different behavioral outcome. The present results, however, do mirror the outcome of our previous study (33), where there was no preference for stimulation of one pain condition over the other. One important note is that within Harris (33), there was a large amount of omission, whereas there were almost neglectable rates of omission in the current study. The unique nature of the current approach creates uncertainty on how to account for the differences between the studies' outcomes, but it is possible that operant-related contingencies and/or strain differences of animals might play a role.

Further, there was no preference to lever-press a particular lever. This was also found in Harris (33), and was thought to be due to a generalization effect such that animals were not able to correctly associate right/left lever-presses to right/left paw stimulation due to the spatial proximity of the levers. In the current protocol, however, the animals were only presented one lever at a time and a light indicator was also presented before the retraction of the lever in an attempt to reduce possible generalization effects. In fact, during the initiation of the light cue, the animals would direct their attention to that lever only suggesting that they “understood” the association between light and lever. Repeated stimulus from both AAP baseline and test days should have created an association between the light, lever, and paw stimulation gave the information known about operant conditioning (48). However, it is difficult to determine the magnitude of the association since there may not always be a perfect association between pain behavior and pain intensity (52).

Theoretically, operant paradigms have the ability to measure changes in behavior and prioritization of drives as a result of disruption by pain. However, there are also possibilities that preclinical chronic pain manipulations have produced weak evidence for the depression of operant responding as a sign of the motivational components of pain or pain affect (53). Taken together, it still remains unclear what exactly the AAP is quantifying since there was no lever-press suppression in the unilateral pain conditions, an outcome that was previously seen in a single lever paradigm by (30) used similar aspects of the AAP. Results from this previous study led us to hypothesize a decrease in lever-pressing in response to the noxious stimuli after induction into the respective pain condition. While we hypothesized that the animals would exhibit a preference to avoid a noxious stimulus, our results indicated a continued approach response, implying an increased salience to food compared to the potential aversiveness associated with either inflammatory or neuropathic pain. We do note, however, that the AAP utilizes the complex integration of both desirable and undesirable effects of the goal of the task, since it is an AAP (32, 47, 54), suggesting there may be changes in decision-making mechanisms depending upon the experience or the motivational context of the current task at hand (55). It has been already suggested that these effects may not effectively translate in describing the quality of pain affect (18) primarily due to the concept that preference for a side does not necessarily equate to levels of aversion. However, the similarity in continued approach behaviors within both pain conditions may not highlight a similarity in aversiveness, but rather a deep complexity between the cognitive and motivational drives associated with hunger and pain, in relation to the ability of various schedules of reinforcement to be able to tease this relationship apart. The presence of pain inevitably elicits the demand for decision-making, but homeostatic function, such as hunger, has been further identified to mediate this relationship in regard to attentional demand spent on each imbalance at a time [LaGraize et al., 2004; (56)]. Thus, highlighting the impact of timing and duration of the response/reinforcer contingency as well as the schedule of reinforcement on behavioral outcomes could help to disentangle the competing cognitive and motivational drives associated with potential dissimilarities in the affective nature of each pain state.

## Limitations

Research seeking to fill the translational gap between preclinical and clinical pain studies have recognized limits within the use of operant techniques. For example, variations in the timing of the response/reinforcer contingency, different schedules of reinforcement, and the challenge of identifying pain episodes within chronic pain states, such as neuropathy, are known to potentially impact behavioral outcomes as measured within operant paradigms (37, 48, 49, 51). While the continued approach behaviors observed in this study highlight important aspects of the relationship between cognitive and motivational drives, there are further limitations, in addition to operant-related constraints, that should be noted. To measure differences in aversion, the degree of the injury inflicted through pain manipulation must be similar across conditions, and the current study did not control for the degree of injury further than the incorporation of a sham surgery condition. While it can be difficult to identify the severity of injury associated with each condition beyond what is observed behaviorally and further control for it across different pain states, we note this as a limitation to be considered in future studies. Additionally, the current study utilized a small number of animals per group (*n* = 6), whereas future studies may serve to benefit from improving the probability of rejecting the null hypothesis by an increase in statistical power.

## Conclusions

The AAP methodology presents a “choice” to animals, which was hypothesized to aid in differentiating between the relative quantity and quality of pain affect associated with inflammatory and neuropathic pain. However, the results from this paradigm seem to suggest that there is no preference for a particular type of pain, less so because the quality of sensation associated with each pain condition is similarly aversive, but more so that the salience of hunger satiation exceeds that of the pain associated with each condition. Thus, these findings suggest differences in clinical and preclinical measures, or at the very least, that our current preclinical behavioral paradigms are not sensitive enough to differentiate the clinically reported affective and cognitive differences across pain conditions, as would be measured through a depression in operant responding. Thus, the need to continue to examine these processes is paramount and will help to guide future studies designed to explore mechanisms of pain relief in clinical populations.

## Data Availability Statement

The raw data supporting the conclusions of this article will be made available by the authors, without undue reservation.

## Ethics Statement

All procedures for this project were approved by the University of Texas at Arlington Institutional Animal Care and Use Committee and were conducted in accordance with the guidelines of the International Association for the Study of Pain.

## Author Contributions

CS designed and performed the experiments and derived the model. CS, CA, and TA analyzed the data. TA and AT assisted with operant measurements. CA helped to carry out SNL surgeries. CS wrote the manuscript in consultation with PF. All authors contributed to the article and approved the submitted version.

## Conflict of Interest

The authors declare that the research was conducted in the absence of any commercial or financial relationships that could be construed as a potential conflict of interest.

## Publisher's Note

All claims expressed in this article are solely those of the authors and do not necessarily represent those of their affiliated organizations, or those of the publisher, the editors and the reviewers. Any product that may be evaluated in this article, or claim that may be made by its manufacturer, is not guaranteed or endorsed by the publisher.
